# Sodium bicarbonate supplementation does not improve elite women's team sport running or field hockey skill performance

**DOI:** 10.14814/phy2.13818

**Published:** 2018-10-14

**Authors:** David Macutkiewicz, Caroline Sunderland

**Affiliations:** ^1^ Sport, Health and Performance Enhancement Research Centre Department of Sports Science Nottingham Trent University Nottingham United Kingdom

**Keywords:** Alkalosis, field‐hockey, football, high‐intensity exercise, prolonged intermittent exercise, supplementation

## Abstract

Team sports, such as field hockey, incorporate high‐intensity repeated sprints, interspersed with low‐intensity running, which can result in acidosis. The aim of the present study was to examine the effect of acute sodium bicarbonate (SB) supplementation on team sport running and skill performance. Eight elite female field hockey players (age 23 ± 5 years, body mass 62.6 ± 8.4 kg, height 1.66 ± 0.05 m) completed three Field Hockey Skill Tests (FHST) interspersed with four sets of the Loughborough Intermittent Shuttle Test (LIST). Prior to exercise, participants were supplemented with capsules equivalent to 0.2 g·kg^−1^ body mass (BM) of a placebo (maltodextrin) or 0.3 g·kg^−1^ BM SB. Field hockey skill performance incorporated overall performance time (PFT), movement time (MT), decision‐making time (DMT), and penalty time (PT). Sprint time (ST), rating of perceived exertion (RPE), blood lactate concentration, bicarbonate anion (${\text{HCO}}_{3}^{-}$) concentration, pH, and base excess were measured at various time points. Data (mean ± SD) were analyzed using a two‐way analysis of variance (ANOVA) with repeated measures, with Hedges *g* effect sizes used to interpret the magnitude of differences. Bicarbonate anion concentration (+5.4 ± 2.6 mmol·L^−1^) and pH (+0.06 ± 0.03) were greater during the bicarbonate trial compared with the placebo (*P* < 0.001). Bicarbonate did not alter PFT (placebo: 87.9 ± 6.9 sec; bicarbonate: 89.0 ± 7.8 sec, *P *=* *0.544, *g* = 0.14), MT, DMT, PT (all *P* > 0.30) or ST (placebo: 2.87 ± 0.12 sec; bicarbonate: 2.86 ± 0.12 sec, *P *=* *0.893, *g* = −0.08). RPE was lower during the SB condition (placebo: 13 ± 2; bicarbonate: 12 ± 2, *P *=* *0.021, *g* = −0.41). Acute ingestion of bicarbonate did not improve sprint or sport‐specific skill performance. Bicarbonate ingestion did result in a lower perception of effort during team‐sport running, which may have performance implications in a competitive match situation.

## Introduction

High‐intensity intermittent exercise requires rapid rates of adenosine triphosphate (ATP) hydrolysis and glycolytic flux, leading to a rise in intracellular, and subsequently, extracellular hydrogen ions (H^+^) (Krustrup et al. 2015). The role of the resulting acidosis in muscular fatigue is much debated and relates to the definition of fatigue, the extent of intracellular pH and changes in the cellular environment, specifically inorganic phosphate and Ca^2+^ (Fitts 2016).

While some findings question the role of H^+^ in causing muscle fatigue (Pedersen et al. 2004), H^+^ accumulation has been reported to contribute to a reduced exercise performance by negatively affecting the perception of effort, ion regulation, enzyme activity, and contractile proteins within the working muscles (Fabiato and Fabiato 1978; Swank and Robertson 1989; Favero et al. 1995; Allen et al. 2003; Girard et al. 2011). This accumulation of H^+^ is likely to be evident during periods of repeated sprint exercise that are observed in team sports and thus may reduce team sport running performance (Krustrup et al. 2015).

Muscle pH has been shown to decrease to 6.96 ± 0.03 compared to resting values (7.24 ± 0.02) after intense periods of fast running and sprinting in the first half of a football match (Krustrup et al. 2006). The ability to counteract these decreases in intracellular (muscle) and extracellular (blood) pH, observed during team sports, may be of great importance if a decline in performance is to be prevented or attenuated. The majority of H^+^ resulting from exercise‐induced metabolic acidosis is buffered by the bicarbonate anion (${\text{HCO}}_{3}^{-}$) (Linderman and Gosselink 1994). Ingesting SB (≥0.3 g·kg^−1^ body mass) elevates the amount of circulating ${\text{HCO}}_{3}^{-}$, increases the bloods buffering capacity and has been shown to protect against acidosis and delay the onset of fatigue during exercise (Costill et al. 1984; Gaitanos et al. 1991; Price et al. 2003; Saunders et al. 2011; Higgins et al. 2013; Krustrup et al. 2015). However, research studies have also demonstrated no such performance improvement (Price and Simons 2010; Peart et al. 2012). The effectiveness of bicarbonate ingestion on sporting performance is impacted by individual variability, training status, timing of ingestion, the changes in ${\text{HCO}}_{3}^{-}$, the exercise task and modality, monocarboxylate transporter activity, and gastrointestinal distress (Saunders et al. 2014; Heibel et al. 2018). Coingestion of SB with food is recommended as reduced gastrointestinal symptoms and concomitant greater ${\text{HCO}}_{3}^{-}$ and pH have been observed (Carr et al. 2011).

In research specifically examining repeated sprint exercise, bicarbonate ingestion has been shown to increase total work done by 5% for cycle sprints (5 × 6 sec, 24 sec recovery) and 2% for treadmill sprints (10 x 6 sec, 30 sec recovery) (Gaitanos et al. 1991; Bishop et al. 2004). Similarly for prolonged intermittent exercise, distance ran increased by 14% in the Yo‐Yo intermittent recovery test level 2 (Yo‐Yo IR2) and relative performance, but not absolute power output, improved during a 30‐min intermittent cycling protocol following preexercise bicarbonate supplementation (Price et al. 2003; Krustrup et al. 2015). In contrast, no performance improvement was evident in highly trained water polo players, during a match‐simulation test incorporating 56 × 10 m maximal sprint swims (Tan et al. 2010). Similarly, in the research by Gaitanos et al. (1991), despite an overall increase in total work done, there was no difference in peak and mean power output of the 10 sprints between placebo and bicarbonate conditions. Thus, the benefits of bicarbonate ingestion on team sport performance are equivocal and warrant further investigation.

The research examining repeated sprint exercise, uses nonsports‐specific exercise modalities and/or fails to examine any skill‐related component, and consequently the practical application of their findings to team sports could be limited. Team sports are characterized by the need to perform bouts of high‐intensity exercise interspersed with short periods of recovery, accompanied by correct decision‐making and technical execution of sport‐specific skills (Burke 1997; Sunderland and Nevill 2005). Technical ability, skill, and accuracy during team sports performance have all been shown to be affected by fatigue resulting from repeated high‐intensity efforts that affect acid–base homeostasis (Davey et al. 2002; Royal et al. 2006; Gabbett 2008, 2016). Davey et al. (2002) have speculated that muscle acidosis impairs skill execution through inhibition of the contractile processes in the muscles responsible for the skill performance. Thus, determining whether bicarbonate ingestion has beneficial effects on skill performance during team sport‐specific exercise warrants investigation.

To date, one study has investigated the effect of bicarbonate ingestion on technique, specifically tennis accuracy, and consistency (Wu et al. 2010). The findings were equivocal, with service and forehand ground stroke consistency improved by ~2−20% following bicarbonate ingestion but service and forehand accuracy, and backhand consistency and accuracy unchanged (Wu et al. 2010). While equivocal, the findings that some aspects of gross motor skills can be improved following bicarbonate supplementation warrant further investigation. Any means of improving skill, accuracy, and technical ability in sport could influence match outcome and should be assessed when investigating the effects of ergogenic aids, such as bicarbonate on team sports performance. Therefore, this study has incorporated a field hockey skill test within the research design.

Women's field hockey is an Olympic sport which is played in over 132 countries across five Continents (FIH, 2018) and has high participation rates. Women's field hockey is a high‐intensity intermittent sport that incorporates periods of high‐speed running and sprinting interspersed with periods of low‐intensity activity (MacLeod et al. 2007). Players cover ~850 m at high speed (>15 km·h^−1^) of which ~230 m is covered by sprinting (>20 km·h^−1^; mean distance 14 m) (Macutkiewicz and Sunderland 2011). During the second half of matches, high‐intensity activity declines (MacLeod et al. 2007) and therefore increasing the blood buffering capacity to buffer the acidosis may enhance performance and is worthy of investigation.

In summary, the research investigating bicarbonate ingestion on team sport performance and skill is limited and equivocal, characterized by a lack of sport‐specific protocols that incorporate relevant skill assessment with almost no focus on elite female team sports athletes. The present study aimed to address these weaknesses. This is the first study to investigate the effects of bicarbonate ingestion on an intermittent running protocol (LIST, Loughborough Intermittent Shuttle Test) that simulates team sport running, and on field hockey skill test performance. It was hypothesized that SB would enhance performance of the repeated maximal sprints incorporated within the LIST and improve performance during the field hockey skill test.

## Materials and Methods

### Participants

Eight elite, female hockey players who played senior, under 21 international hockey and/or in the National English Hockey League volunteered as participants for the study. Mean (±SD) age, body mass, height, estimated maximal oxygen uptake ($\dot{V}O_{2}$ max) from the multistage fitness test (Ramsbottom et al. 1988) were 23 ± 5 years, 62.6 ± 8.4 kg, 1.66 ± 0.05 m, and 50.5 ± 3.1 mL·kg^−1^·min^−1^, respectively. Seven of the participants had normal menstrual cycles and one had been taking an oral contraceptive for more than a year. All participants were involved in regular field hockey training (at least five times a week), which included pitch sessions and conditioning sessions, and fully informed about the rationale for the investigation and all experimental procedures to be undertaken. After completion of a health screen questionnaire, all participants’ provided their written informed consent for the study, which had institutional ethics approval.

### Experimental design

Participants reported to the laboratory on five separate occasions, three preliminary visits and two main trials. The first session involved a progressive multistage fitness test (Ramsbottom et al. 1988) to determine estimated $\dot{V}O_{2}$ max and running speed for the Loughborough Intermittent Shuttle Test (Nicholas et al. 2000). During the subsequent two visits, participants performed 1–2 blocks of the LIST Part A and/or the Field Hockey Skill Test as habituation to the key outcome measurements and running required during the experimental protocol. Each participant completed two experimental trials following the ingestion of either SB or a placebo. Experimental trials were single‐blinded and conducted in a randomized crossover design.

## Experimental procedures

The two experimental trials were conducted during the follicular phase of the menstrual cycle (days 4–14) or during days 5–20 of resuming the oral contraceptive pill, which was verified by the measurement of plasma progesterone concentrations. Resting progesterone concentrations were similar between trials (placebo: 0.48 ± 0.16 nmol·l^−1^; bicarbonate: 0.51 ± 0.31 nmol·l^−1^, *P *=* *0.789, *d* = 0.13). A minimum of 5 days elapsed between the SB and placebo trials.

Participants had not taken any supplement in the 3 months prior to the study and had not taken *β*‐alanine for at least 6 months. Participants were asked to record their food consumption for 24 h prior to the initial experimental trial which was replicated and verbally verified before the subsequent trial. Participants abstained from intense exercise, alcohol and any caffeine containing products for 48 h prior to each trial.

Participants were able to consume water ad libitum throughout the trial. Pre and postexercise body mass (Seca 873; Hamburg, Germany) was recorded allowing sweat loss to be estimated after adjustments for fluid consumption and urinary losses were made. In order to determine hydration status, participants provided a baseline morning urine sample to ensure euhydration prior to exercise (urine osmolality >900 mOsmol·kg^−1^; Osmomat 030, Gonotec, Berlin, Germany (Shirreffs and Maughan 1998)). Temperature and humidity were measured throughout the exercise protocol using a whirling hygrometer (Casella, London, UK).

### Loughborough intermittent shuttle test (LIST)

The LIST Part A, (Nicholas et al. 2000) modified by Sunderland and Nevill (2005) to replicate the physiological demands of female field hockey, requires participants to exercise over a 20‐m distance repeating a 3 × 20 m walk, 1 × 15 m sprint, 3 × 20 m run (~85% $\dot{V}O_{2}$ max) and 3 × 20 m jog (~50% $\dot{V}O_{2}$ max) pattern 11 times approximating 15 min, followed by a 3‐min rest period. This pattern of exercise constitutes one set of the LIST. Participants performed four sets of the LIST in total.

### Field hockey skill test (FHST)

The skill test was performed on an artificial synthetic water‐based turf similar to the type of turf used for international and national matches. A detailed description of procedure for the FHST can be found in Sunderland et al. (2006). The skill test involves dribbling, passing, and shooting at a randomized side of the goal determined by the switching on of a light, once an infrared light beam is broken. The time taken between the participants breaking the light beam to the ball hitting the backboard was termed the “decision‐making time” as it incorporates the players’ responses and decisions within the time taken. Immediately after shooting, participants ran to the start line and repeated the test a further five times. When the participants returned to the start line after the sixth and final attempt, the timer was stopped and this was termed “movement time”. An additional 2‐sec penalty was added to the movement time if the participant missed a target, kicked the ball, or hit a cone. The movement time was then recalculated to form “performance time” (movement time + penalty time). The FHST has previously been reported to be reliable with a mean difference and 95% confidence limits of 0.0 ± 1.0% for overall performance time and a mean difference and 95% confidence limits of 0.0 ± 1.0% for decision‐making time (Sunderland et al. 2006). For overall performance time, the smallest worthwhile change is 2 sec (Sunderland et al. 2006).

### Main trial

**Figure 1:**
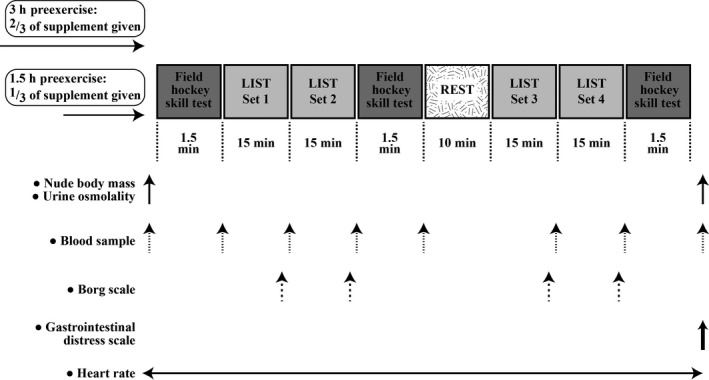
Schematic of the main trial protocol.

Figure 1 is a schematic of the main trial protocol. Participants reported to the laboratory at the same time of day, in the morning, to control for circadian influences, following an overnight fast (Winget et al. 1985). Stature (Seca, Hamburg, Germany), nude body mass (GFK 150, Adam Equipment Co. Ltd, Milton Keynes, UK) baseline blood, urine osmolality, and FHST performances were recorded. Following a standardized breakfast of three slices of white toast and jam participants ingested either ^2^/_3_ of SB (0.3 g·kg^−1^ body mass) or placebo (maltodextrin, 0.2 g·kg^−1^ body mass) in opaque gelatin capsules with water consumed ad libitum. Participants rested for 90 min, then consumed with a standardized cereal bar the remaining ^1^/_3_ of the supplement and rested for a further 90 min. While the dose used for supplementation was within the suggested optimal range, a split dose protocol has been previously used (Swank and Robertson 1989; Sale et al. 2010; Saunders et al. 2014) and was employed in an attempt to minimize any gastrointestinal discomfort. Preexercise body mass and blood were taken before performing a standardized warm‐up which consisted of levels 1 and 2 of the progressive multistage fitness test, 4–5 × 15 m sprints where the participants were instructed to build the intensity of the sprints such that sprints 4 and 5 were maximal efforts and 2 min stick and ball practice on the synthetic turf. Participants then performed three FHST and four sets of the LIST; one FHST was completed before the start of the LIST and then repeated after set 2 and again after set four of the LIST. The participants rested passively for a total of 10 min after the first two sets to mimic half‐time in a competitive match. The 15 m sprint times were recorded using a wireless photocell timing system (Brower Timing Systems, Model IRD‐T175, Utah) and participants were verbally encouraged to perform maximally during the 15 m sprints and the FHST.

Heart rate was monitored continuously (5s sampling, Polar team system, Finland). Rating of perceived exertion (RPE) using the Borg scale (Borg 1982) was recorded before the eleventh sprint in each exercise set. Blood samples were collected from the participants between the sets of exercise and after each FHST. Gastrointestinal distress (stomach ache and sickness) and headache were determined at the end of each trial via the administration of a short questionnaire which incorporated scales. The scales ranged from 0 to 10 and contained descriptors at 0, 3, 6, 9, and 10. For stomach ache, the descriptors were *none at all*,* dull ache on and off*,* moderate continuous*,* severe continuous*, and *severe doubled up* and those for sickness were *not at all*,* slightly*,* quite*,* very*, and *throwing up. F*or headache, the descriptors were *none at all*,* dull ache on and off*,* moderate continuous*,* severe continuous*,* and searing pain*.

### Blood sampling and analysis

Ten milliliter of venous blood was taken from an indwelling cannula at rest (baseline), before the first skill test, between each set of the LIST and after the FHST at half‐time and the end of exercise. Blood was dispensed into 1 × 5 mL chilled Lithium‐Heparin tube and 1 × 5 mL plain, anticoagulant‐free serum tubes. Blood was analyzed for blood glucose and lactate concentrations using a fully automated machine (YSI, Stat 2300 Plus, Ohio) and for pH using a bench top blood gas analyzer (Radiometer Ltd, ABL 700, Crawley, UK). Bicarbonate (${\text{HCO}}_{3}^{-}$) and base excess were calculated using the Henderson‐Hasselbach and Siggaard‐Anderson equations.

The remaining blood was then centrifuged at 1450 *g* for 10 min at 4°C. After centrifuging, the supernatant was removed, divided into aliquots of ~1.0 mL, one aliquot was immediately used for the determination of serum osmolality via a cryoscopic osmometer (Osmomat 030, Gonotec, Berlin, Germany) and remaining aliquots frozen at −80°C until the analyses were performed. Plasma concentrations of progesterone were determined via enzyme linked immunoassay (DRG Instruments, GmbH, Marburg Germany).

### Statistical analyses

Descriptive data are reported as mean ± standard deviation and data were checked for normality by visual inspection of the Q‐Q plot and histograms. A two‐way analysis of variance (ANOVA) with repeated measures was used to determine differences in all variables across conditions (placebo, SB) and time. Variables analyzed were skill performance times, sprint times, RPE, and blood responses. Data were checked for sphericity using Mauchly's test, where sphericity was violated a Greenhouse‐Geisser correction was used. A Tukey's post hoc was used to determine where differences lay (Field 2009). A paired *t*‐test was used to determine any differences in environmental conditions, hydration status, body mass, fluid consumption, and sweat rate. Effect sizes were calculated using Hedges *g* (95% confidence intervals) (Cohen 1988) and interpreted using the following thresholds: <0.2 = trivial effect; 0.2–0.5 = small effect; 0.5–0.8 = moderate effect and >0.8 = large effect (Cohen 1992). Significance was set at *P *<* *0.05.

## Results

### Environmental conditions

No differences in ambient temperature (placebo: 20.1 ± 2.7°C; bicarbonate: 20.3 ± 2.9°C; *P *=* *0.873, *g* = 0.07 (−0.91, 1.05) trivial effect) or relative humidity (placebo: 55.5 ± 6.9%; bicarbonate: 55.8 ± 6.8%; *P *=* *0.975, *g* = 0.04 (−0.94, 1.02) trivial effect) were observed between the two trials.

### The field hockey skill test (FHST)

**Figure 2:**
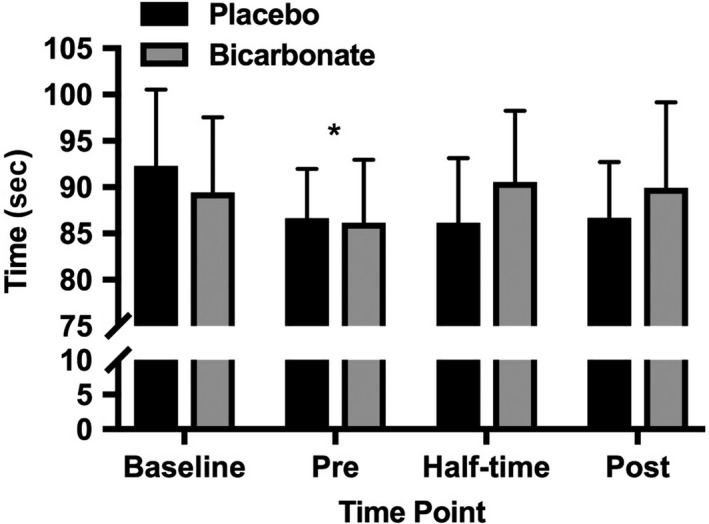
Mean ± SD performance time of the Field Hockey Skill Tests for the placebo and bicarbonate trials, (main effect time *P *<* *0.05; condition × time interaction *P *<* *0.05, *P < 0.05 from baseline).

No differences were found between conditions for mean performance time (placebo: 87.9 ± 6.9 sec; bicarbonate: 89.0 ± 7.8 sec, *P *=* *0.544, *g* = 0.14 (−0.84, 1.12) trivial; Fig. 2). However, a time effect (*P *=* *0.012) and an interaction between condition and time were observed (*P *=* *0.038, Fig. 2). The preexercise skill test was quicker than the baseline (*P* < 0.05). There were no significant post hoc differences after the interaction suggested significance.

No main effect for condition or time was observed for movement time (placebo: 83.00 ± 5.97 sec; bicarbonate: 83.24 ± 6.15 sec, *P *=* *0.86, *g* = 0.04 (−0.94, 1.02) trivial effect), penalty time (placebo 4.9 ± 2.7 sec; bicarbonate: 5.8 ± 3.5 sec, *P *=* *0.28, *g* = 0.27 (−0.71, 1.26) small effect) or decision‐making time (placebo: 4.54 ± 0.11 sec; bicarbonate: 4.52 ± 0.10 sec, *P *=* *0.81, *g* = −0.18 (−1.16, 0.80) trivial effect; Table 1). A condition x time interaction was found for decision‐making time (*P *=* *0.025), demonstrating quicker decision‐making during the placebo condition at half‐time compared with baseline (post hoc *P *=* *0.019, *g* = −1.67 (−2.81, −0.53) large effect).

**Table 1:** Mean ± SD movement time, decision‐making time, and penalty time during the placebo and sodium bicarbonate conditions

	Baseline	Pre FHST	HT	Post FHST
Movement time (sec)				
Placebo	85.5 ± 6.1	81.9 ± 4.8	82.6 ± 6.2	81.9 ± 7.0
NaHCO_3_	83.9 ± 6.2	81.4 ± 4.1	83.3 ± 6.9	84.3 ± 7.7
Decision‐making time (sec)				
Placebo	4.96 ± 0.46	4.47 ± 0.43	4.28 ± 0.29*	4.45 ± 0.55
NaHCO_3_	4.43 ± 0.34	4.55 ± 0.35	4.55 ± 0.35	4.69 ± 0.50
Penalty time (sec)				
Placebo	6.8 ± 3.0	4.8 ± 1.8	3.5 ± 1.8	4.8 ± 3.4
NaHCO_3_	5.5 ± 3.7	4.8 ± 3.8	7.3 ± 3.7	5.6 ± 2.7

* Different to placebo baseline *P *<* *0.05.

NaHCO_3_, sodium bicarbonate; FHST, Field Hockey Skill Test; HT, Half‐time.

### The Loughborough intermittent shuttle test (LIST)

**Figure 3:**
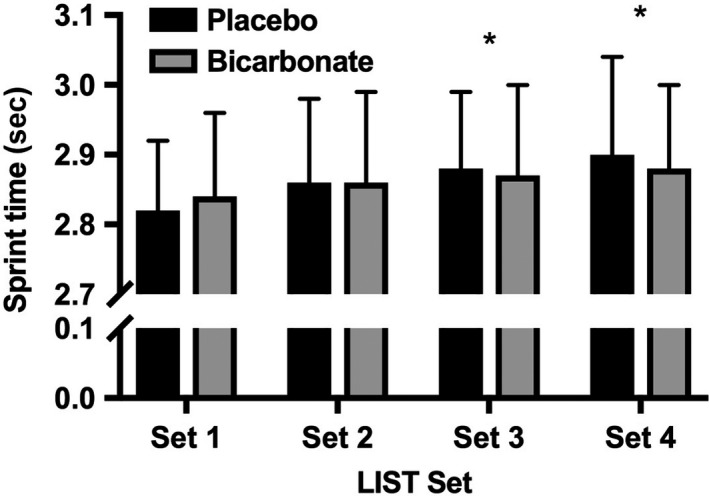
Mean ± SD sprint times during the Loughborough Intermittent Shuttle Test for the placebo and bicarbonate trials, (main effect time *P *<* *0.001, *P < 0.01 from Set 1).

The mean sprint time for the placebo and bicarbonate conditions was not different and was 2.87 ± 0.12 sec and 2.86 ± 0.12 sec, respectively (*P* = 0.893, *g* = −0.08 (−1.06, 0.90) trivial effect; Fig. 3). Sprint time declined with time, (time effect *P *≤* *0.001) and closer inspection revealed that mean sprint times for Set 3 (2.88 ± 0.12 sec, *P *=* *0.004, *g* = 0.41 (−0.58, 1.40) small effect) and Set 4 (2.89 ± 0.13 sec, *P* ≤ 0.001, *g* = 0.51 (−0.52, 1.46) small effect) were greater than Set 1 (2.83 ± 0.11 sec).

### Heart rate, RPE, and blood responses

No condition effect was observed in the heart rate responses to the LIST and FHST (Table 2). A time effect (*P *≤* *0.001) revealed that heart rate was higher for all time points once participant's commenced the LIST than the baseline FHST (*P *<* *0.05).

**Table 2:** Mean ± SD heart rate, pH, lactate, glucose, ${\text{HCO}}_{3}^{-}$, and base excess response during the placebo and NaHCO_3_ conditions

	Baseline	Pre FHST	Set 1	Set 2	HT	Set 3	Set 4	Post FHST
Heart rate (beats·min^−1^)†								
Placebo	160 ± 9	165 ± 10	168 ± 10	171 ± 11	173 ± 10	168 ± 11	170 ± 10	170 ± 11
NaHCO_3_	159 ± 11	169 ± 9	166 ± 12	167 ± 14	166 ± 15	165 ± 12	167 ± 13	168 ± 10
pH# ^,^ † ^,^ ‡								
Placebo	7.403 ± 0.015	7.400 ± 0.028	7.379 ± 0.046	7.395 ± 0.048	7.346 ± 0.074	7.400 ± 0.032	7.401 ± 0.041	7.339 ± 0.063
NaHCO_3_	7.407 ± 0.012	7.480 ± 0.018*	7.454 ± 0.028*	7.466 ± 0.038*	7.397 ± 0.060*	7.471 ± 0.030*	7.465 ± 0.028*	7.408 ± 0.050*
Lactate (mmol·L^−1^)†								
Placebo	1.0 ± 0.3	1.1 ± 0.2	4.6 ± 1.6	4.8 ± 1.1	7.2 ± 1.8	4.7 ± 1.3	4.9 ± 1.5	7.7 ± 1.7
NaHCO_3_	1.0 ± 0.2	1.1 ± 0.4	4.9 ± 2.1	5.1 ± 1.4	8.2 ± 2.4	5.1 ± 1.6	5.5 ± 1.6	7.5 ± 2.7
Glucose (mmol·L^−1^)†								
Placebo	3.7 ± 0.6	4.0 ± 0.4	4.7 ± 1.0	5.1 ± 1.3	4.8 ± 1.2	4.9 ± 1.2	5.5 ± 1.3	5.2 ± 1.2
NaHCO_3_	4.1 ± 0.6	3.7 ± 0.9	4.4 ± 1.7	5.2 ± 1.2	4.8 ± 1.3	4.9 ± 1.4	5.2 ± 1.0	4.7 ± 1.11
${\text{HCO}}_{3}^{-}$ (mmol·L^−1^)# ^,^ † ^,^ ‡								
Placebo	25.7 ± 1.2	25.4 ± 1.7	18.8 ± 1.8	19.5 ± 2.6	15.9 ± 2.0	20.0 ± 2.1	19.4 ± 2.49	16.8 ± 1.6
NaHCO_3_	25.9 ± 1.4	31.4 ± 1.4*	24.3 ± 1.9*	25.6 ± 2.1*	22.0 ± 2.3*	25.4 ± 2.4*	25.6 ± 3.1*	21.7 ± 2.9*
Base excess (mmol·L^−1^)# ^,^ † ^,^ ‡								
Placebo	1.4 ± 1.0	1.1 ± 1.4	−5.4 ± 2.1	−4.6 ± 2.7	−8.8 ± 2.5	−4.1 ± 2.1	−4.6 ± 2.5	−7.9 ± 1.8
NaHCO_3_	1.6 ± 1.2*	7.6 ± 1.3*	0.7 ± 1.9*	2.1 ± 1.9*	−2.2 ± 2.7*	1.9 ± 2.4*	2.0 ± 2.9*	−2.3 ± 3.1*

NaHCO_3_, sodium bicarbonate; Pre FHST, The first Field Hockey Skill Test prior to running; HT, Half‐time; Post FHST, The final Field Hockey Skill Test at the end of running.

^#^main effect condition *P *<* *0.001, ^†^main effect time *P *<* *0.05; ^‡^condition × time interaction *P *<* *0.001; *P < 0.01 from placebo at equivalent time point.

**Figure 4:**
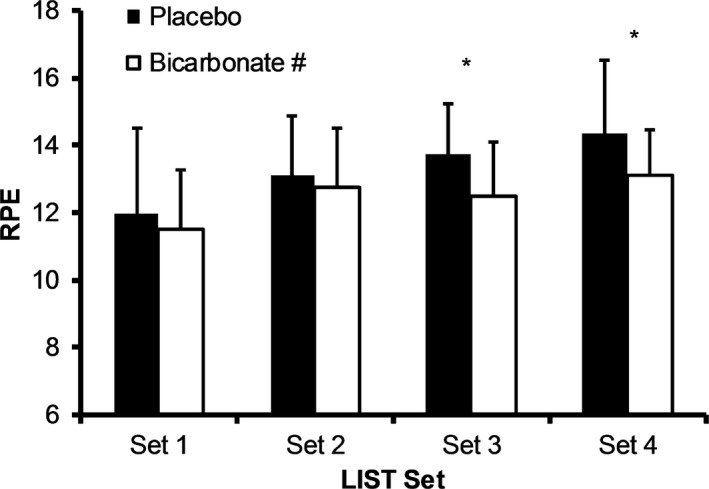
Mean ± SD rating of perceived exertion during the Loughborough Intermittent Shuttle Test for the placebo and bicarbonate trials, (^#^main effect condition *P *<* *0.05 bicarbonate lower than placebo, main effect time *P *<* *0.001, *P < 0.01 from Set 1).

RPE was lower during the bicarbonate condition (*P *=* *0.021, *g* = −0.41 [−1.40, 0.58] small effect Fig. 4). RPE increased with time (*P *=* *0.004) and was higher during Set 3 (13 ± 2, *P *=* *0.04, *g* = 0.69 [−0.32, 1.70] moderate effect) and Set 4 (14 ± 2, *P *=* *0.002, *g* = 0.96 [−0.08, 1.99] large effect) than Set 1 (12 ± 2).

No differences in blood lactate or blood glucose concentrations were observed between the placebo and bicarbonate conditions, a time effect was, however, found in blood lactate (*P *<* *0.001, Table 2) and blood glucose (*P *<* *0.001, Table 2).

A main effect for condition (pH *P *<* *0.001; ${\text{HCO}}_{3}^{-}$ *P *≤* *0.001; Base excess *P *≤* *0.001), time (*P *≤* *0.05) and an interaction (*P *≤* *0.001) were found for pH, ${\text{HCO}}_{3}^{-}$ and base excess (Table 2). After the ingestion of bicarbonate, pH, ${\text{HCO}}_{3}^{-}$ and base excess were greater than in the placebo trial (*P *≤* *0.001).

### Participant hydration, body mass, fluid consumption, and sweat rates

Mean urine osmolalities below 900 mOsmol·kg^−1^ (Shirreffs and Maughan 1998) indicated that participants reported to the laboratory in a euhydrated state and remained euhydrated prior to commencing exercise during both trials (morning: placebo 435 ± 280 mOsmol·kg^−1^, bicarbonate 500 ± 271; preexercise: placebo 143 ± 51 mOsmol·kg^−1^; bicarbonate: 530 ± 145 mOsmol·kg^−1^). Participants lost greater body mass during the placebo trial (placebo 0.56 ± 0.29 kg, SB 0.36 ± 0.28 kg *P *=* *0.04, *g* = −0.66 [−1.67, 0.34] moderate effect). No difference was observed in the volume of water consumed between conditions (placebo 652 ± 305 mL; bicarbonate: 824 ± 299 mL, *P *=* *0.156, *g* = 0.54 [−0.46, 1.54] moderate effect). After correcting for fluid consumption and urinary loss, mean sweat loss was 1216 ± 375 mL for the placebo trial and 1195 ± 344 mL for the bicarbonate trial (*P *=* *0.636, *g* = −0.06 [−1.04, 0.93] trivial effect) and sweat rates were 0.82 ± 0.25 L·h^−1^ (placebo) and 0.81 ± 0.23 L·h^−1^ (bicarbonate, *P *=* *0.640, *g* = −0.04 [−1.02, 0.94] trivial effect).

### Gastrointestinal distress

All participants reported no symptoms of gastrointestinal distress or headache (all scores = 0).

## Discussion

The present study investigated the influence of acute ingestion of bicarbonate on intermittent running (which simulates the physical demands of team sport type activities) and on hockey skill performance. Bicarbonate did not produce any performance improvements, with no meaningful differences observed in sprint performance during the LIST, or in performance time, movement time or decision‐making time during the FHST. Bicarbonate ingestion reduced ratings of perceived exertion and body mass loss following exercise. Blood ${\text{HCO}}_{3}^{-}$ concentration, pH, and base excess were greater following acute ingestion of bicarbonate than the placebo trial.

It is possible that the combined physical and skill demands of the LIST and FHST did not pose enough of an anaerobic or acid–base balance disturbance on the body to overly stress the body's natural buffering capacity. Blood pH and ${\text{HCO}}_{3}^{-}$ during both the LIST and FHST remained high in both trials, while blood lactate concentrations were lower in both conditions compared with concentrations reported in previous research that has found improved performance following bicarbonate ingestion (Costill et al. 1984; Bouissou et al. 1988; Price et al. 2003). Performance improvements following bicarbonate ingestion have been associated with a decline in blood pH to below 7.2, blood lactate values in excess of 10 mmol·L^−1^ and a decline in HCO_3_ in the range of 10–15 mmol·L^−1^ (Costill et al. 1984; Bouissou et al. 1988; Price et al. 2003). In the present study however, after the final skill test, pH values of 7.34 and 7.41 for the placebo and bicarbonate conditions were considerably higher than the values associated with the performance benefits described above. Furthermore, blood lactate concentrations of 7.69 (placebo) and 7.46 (bicarbonate) mmol·L^−1^, and a decline of 8.7 (placebo) and 9.7 mmol·L^−1^ (bicarbonate) in ${\text{HCO}}_{3}^{-}$, were also relatively small responses in comparison to the >10 mmol·L^−1^ blood lactate concentration and declines in HCO_3_ of 10–15 mmol·L^−1^ were observed in studies where performance benefits accrued. This would suggest that in the present study performance decrements due to H^+^ accumulation were unlikely, and the extra buffering capacity provided by the preexercise bicarbonate ingestion remained unnecessary and consequently ineffectual.

In contrast to the present study, bicarbonate supplementation has been shown to result in a 14% improvement in Yo‐Yo IR2 capacity and the maintenance of sprint performance during prolonged intermittent exercise (Price et al. 2003; Krustrup et al. 2015). The level of acidosis experienced during Yo‐Yo IR2 (7.04 placebo and 7.19 bicarbonate [Krustrup et al. 2015]) and intermittent protocol employed by Price et al. (2003) (7.21 placebo and 7.29 bicarbonate), however, was greater than the present study and in line with previous research. Price et al. (2003) utilized 14 sec sprints, a duration which is considerably greater than the 2–4 sec seen in team sports (MacLeod et al. 2007; Macutkiewicz and Sunderland 2011; Johnston et al. 2014; Sunderland and Edwards 2017) and therefore may place a greater challenge to acid–base balance and a greater demand on buffering capacity.

The findings of the present study, that skill and sprint performance were unchanged (both trivial effects), are in agreement with previous research (Bishop and Claudius 2005; Price and Simons 2010). Despite finding greater work done in 7 of 18 “second half” 4‐sec cycle sprints, Bishop and Claudius (2005) reported no meaningful difference in total work or mean power output during a prolonged intermittent sprint test. The protocol employed by Bishop and Claudius (2005), equally failed to cause a fall in pH below 7.2 (7.38 placebo, 7.50 bicarbonate) and resulted in lower peak lactates (3.2 and 4.0 mmol·L^−1^) than expected, suggesting insufficient demand on the bodies buffering capacity for bicarbonate ingestion to be of benefit. Similarly in elite water polo players, performance was not improved during 56 × 10 m maximal sprint swims, with pH values >7.2 (Tan et al. 2010). In the only other study to incorporate technique performance, in the form of tennis serves and groundstrokes, Wu et al. (2010) reported some improvements in consistency scores, but these were only for the serve and forehand with no beneficial effects reported for accuracy (pH = 7.37). These findings seem to confirm that acid–base balance has to be substantially disturbed before preexercise bicarbonate ingestion might be of benefit. Intermittent exercise and skill performance, typical of many sports, may not elicit the degree of disturbance necessary to benefit from bicarbonate ingestion.

The participants in the present study were elite field hockey players and therefore their training status may have contributed to the lack of a performance improvement. In a meta‐analysis examining bicarbonate use for athletic performance, Peart et al. (2012) compared research from trained participants, defined as those whose ‘training plan is relevant for the respective exercise task’ and untrained, defined as ‘healthy, active and recreationally trained’ (Peart et al. 2012). The analysis revealed a lower effect size, and hence benefit of bicarbonate in studies employing trained participants, perhaps due to an improved muscle buffering capacity and an increased density of monocarboxylate transporter proteins in trained athletes (Peart et al. 2012). The lack of a performance improvement in the elite female field hockey players in the present study and that observed in elite female water polo players (Tan et al. 2010), supports the assertion that during team sports, elite players may not benefit from bicarbonate supplementation because their training status confers benefits that cannot be enhanced by bicarbonate supplementation. The elite nature of the participants in the present study limited the sample size and thus may have meant the study was underpowered. However, the overall change in performance in the skill test was −2.8%, which suggests performance was not improved by bicarbonate ingestion.

Perception of effort during the LIST was lower during the bicarbonate condition (small effect). It has been suggested that perception of effort during high‐intensity intermittent exercise is related to the buffering capacity of the blood (Swank and Robertson 1989), with alkalosis, through the ingestion of bicarbonate, attenuating ratings of perceived exertion during high‐intensity continuous and intermittent exercise (Robertson et al. 1986; Swank and Robertson 1989; Higgins et al. 2013; Krustrup et al. 2015). A lowered RPE may represent a central affect that results in less negative feedback from the muscle and a greater maintenance of voluntary activation (Krustrup et al. 2015). Despite lower perceived exertion ratings being observed in the present study during the bicarbonate condition, this did not correspond with an improvement in sprint or FHST performance. However, during actual match play where sprint efforts and skill challenges are not controlled, as in the current study, an attenuated RPE response as a result of bicarbonate ingestion may enable performers to produce higher intensity efforts, or such efforts more frequently, and maintain skill.

In the present study, there was a greater body mass loss (moderate effect) and lower urine osmolality preexercise in the placebo condition than the bicarbonate condition demonstrating increased total body water content following ingestion of bicarbonate. To date, the research incorporating prolonged intermittent exercise and bicarbonate ingestion has not reported body mass changes and thus comparisons with other research reports are not possible (Price et al. 2003; Tan et al. 2010; Wu et al. 2010; Krustrup et al. 2015). One study involving lightweight rowers did measure body mass changes and urine losses in the 2 h prior to exercise and found attenuated urine loss (medium effect size) with bicarbonate ingestion (Kupcis et al. 2012). It should be noted, however that alongside the bicarbonate ingestion, participants in the Kupcis et al. (2012) study did ingest a recovery solution containing a substantial sodium content (31.8 mg Na^+^·kg^−1^ body mass) which would have stimulated rehydration, thus may have limited the changes in body mass. The likely mechanism for the smaller reduction in body mass when ingesting bicarbonate is the retention of fluid in response to the sodium intake (Lindinger et al. 2000; Mora‐Rodriguez and Hamouti 2012). Plasma volume, intracellular and extracellular fluid, and thus total body water are all increased following SB ingestion (Lindinger et al. 2000). The sodium intake in the bicarbonate condition was not matched in the placebo and thus is a limitation. However, even though sodium assists with fluid retention and thus may alleviate cardiovascular and thermoregulatory responses (Mora‐Rodriguez and Hamouti 2012), in the thermoneutral conditions with ad libitum fluid intake, the additional sodium intake did not have a performance benefit. This was demonstrated during the repeated sprinting and the skill performance test by the similar heart rate response in both trials. In elite athletes in competitive situations, several factors can influence, and often reduce the opportunities to drink (Garth and Burke 2013). With bicarbonate ingestion, the gastrointestinal tract may act as a reservoir and thus assist with the maintenance of extracellular fluid volume during prolonged exercise (Lindinger et al. 2000). Therefore, bicarbonate ingestion prior to exercise could have beneficial effects during team sports performance, particularly in hot environments by helping to maintain fluid balance as well as acting as a buffer.

Recent research has introduced the concept of responders and nonresponders when using bicarbonate as an ergogenic aid (Saunders et al. 2014). Saunders et al. (2014) demonstrated that differences in the preexercise to postexercise change in blood pH, ${\text{HCO}}_{3}^{-}$ and base excess exists between individuals who improved total work done during two cycling capacity tests at 110% of maximum power and those who did not improve. The sample size of the current study was insufficient to determine responders versus nonresponders, but this may be an explanatory factor for the improved perception of effort without a concomitant performance improvement. In the present study, only two participants improved overall skill performance with bicarbonate ingestion. In addition, dosing strategy in terms of amount and timing of bicarbonate ingestion has recently received much attention (Jones et al. 2016; Gough et al. 2018), with a suggestion that time to peak ${\text{HCO}}_{3}^{-}$ be used to maximize performance benefits (Gough et al. 2018). In the present study, time to peak ${\text{HCO}}_{3}^{-}$ was not used, however during the current protocol lasting ~90 min in total, where individual differences in bicarbonate appearance and clearance from the blood will occur, this seems less important. Of note, is that ${\text{HCO}}_{3}^{-}$ was higher throughout the trial with bicarbonate ingestion.

## Conclusions

The present study was the first to investigate the effect of bicarbonate ingestion on team sport skill, specifically field hockey skill. The findings suggest that, in elite female field hockey players, acute ingestion of bicarbonate does not improve prolonged intermittent or sport‐specific skill performance designed to simulate hockey performance, despite decreasing the perception of effort. In summary, in the present match simulation, there is no evidence of a benefit of bicarbonate ingestion on hockey skill performance, likely due to a lack of hydrogen cation accumulation and enhanced buffering of the elite participants. However, there was a lower perception of effort with bicarbonate, which may be an important change affecting performance during matches.
